# Long-term kinetics of Zika virus RNA and antibodies in body fluids of a vasectomized traveller returning from Martinique: a case report

**DOI:** 10.1186/s12879-016-2123-9

**Published:** 2017-01-10

**Authors:** Guenter Froeschl, Kristina Huber, Frank von Sonnenburg, Hans-Dieter Nothdurft, Gisela Bretzel, Michael Hoelscher, Lothar Zoeller, Matthias Trottmann, Francisco Pan-Montojo, Gerhard Dobler, Silke Woelfel

**Affiliations:** 1 Division of Infectious Diseases and Tropical Medicine, Medical Center of the University of Munich (LMU), Leopoldstr. 5, 80802 Munich, Germany; 2 German Centre for Infection Research (DZIF), Partner Site Munich, Munich, 80802 Germany; 3 Bundeswehr Institute of Microbiology (BwIM), Neuherbergstrasse 11, 80937 Munich, Germany; 4 Department of Urology, Medical Center of the University of Munich (LMU), Marchioninistr. 15, 81377 Munich, Germany; 5 Department of Neurology, Medical Center of the University of Munich (LMU), Marchioninistr. 15, 81377 Munich, Germany

**Keywords:** Zika virus, Whole blood, Ejaculate, Vasectomy, Cell culture, Phylogeny, Case report

## Abstract

**Background:**

The magnitude of the current Zika virus (ZIKV) epidemic has led to a declaration of a Public Health Emergency of International Concern by the WHO. Findings of viable viral particles in semen for several weeks are corroborating reports of sexual transmission of ZIKV. Serious consequences of a positive diagnostic result particularly in the pregnant patient are calling for precise diagnostic tools also at later time points after infection. Currently, recommendations suggest a diagnostic period of direct viral detection of 5 to 7 days after onset of symptoms in serum or plasma, and up to 3 weeks in urine samples.

**Case presentation:**

A vasectomized 41-year-old German returning from Martinique presented at the outpatient clinic of the Department for Infectious Diseases and Tropical Medicine, Munich, with subfebrile temperature, rash, malaise, severe retro-orbital pain and occipital lymphadenopathy. The main complaints resolved after ten days without specific treatment. We are reporting on clinical course and results of direct and indirect detection methods of ZIKV in different sample types including whole blood, ejaculate, urine, serum, plasma and saliva samples up to 119 days post symptom onset. Ejaculate samples remained PCR positive for ZIKV until day 77, whole blood samples until day 101.

**Conclusions:**

The case presentation adds to the still limited knowledge of kinetics of detection of ZIKV by direct as well as indirect methods. Here, a complete data set including results from PCR, serology and cell culture is provided allowing an improved evaluation of optimum diagnostic periods for testing a variety of sample types. Moreover, a high viral load of ZIKV RNA was detected in ejaculate of the vasectomized patient. This finding sheds new light on the possible localizations of ZIKV replication in the human male reproductive tract.

## Background

Since its discovery in 1947 until 2007 only single cases of human Zika virus (ZIKV) infections with mostly benign presentations in Africa and South East Asia have been reported [1]. The disease entity has not received particular attention until recently, and in the 2010 edition of Harrison’s Infectious Diseases for example the infection is not covered at all [2]. After smaller outbreaks on the Yap islands and in French Polynesia, an unprecedented epidemic of ZIKV infections has been observed in South America and the Caribbean since 2014, with an estimated number of more than 1 million cases by now in Brazil alone [3]. The concomitant appearance of neonatal neurological malformations and cases of Guillain-Barré-syndrome in regions affected by the ZIKV epidemic have led to a declaration of a Public Health Emergency of International Concern by the WHO on 01 February 2016 [4]. The potentially severe consequences of a positive diagnosis in pregnant women are contrasted by weaknesses in the diagnostic algorithms currently in use. In patients presenting symptoms that are suggestive of ZIKV infection (fever and/or rash, plus arthralgia or arthritis or conjunctivitis) confirmation of the infection is based on serological testing of serum samples for IgM and IgG antibodies against ZIKV and on nucleic acid amplification tests in serum and urine samples [5]. Serological diagnosis is regularly hampered by cross-reactivities with antibodies against other flaviviruses resulting from prior infections or vaccinations. For the nucleic acid amplifications tests, the reportedly brief diagnostic period of only 5 to 7 days when using standard samples such as plasma and serum reduces the diagnostic sensitivity of these very specific methods [6]. However, the use of urine samples may extend the detection period to up to 3 weeks [5]. Reports of sexual transmission of ZIKV and the detection of ZIKV RNA in ejaculate beyond 60 days post symptom onset make ejaculate a promising specimen for late diagnosis of Zika in male patients [7, 8]. The location and mechanisms of ZIKV persistence in the male reproductive organ are not yet clearly understood. Moreover, it is still unclear whether the prolonged detection in ejaculate is a general phenomenon in male patients as up to now only a limited number of cases have been investigated.

That in whole blood samples the virus may be detectable for longer periods of time as compared to serum samples has previously been reported for other flaviviruses such as dengue virus (DENV) [9] and West Nile virus (WNV) [10], and most recently also for ZIKV [11].

In the human case reported by us now ZIKV was detected for a long period in various sample types up to 101 days post symptom onset. In addition, ZIKV could be successfully cultured from ejaculate.

## Case presentation

### Clinical history and physical findings

**Fig. 1:**
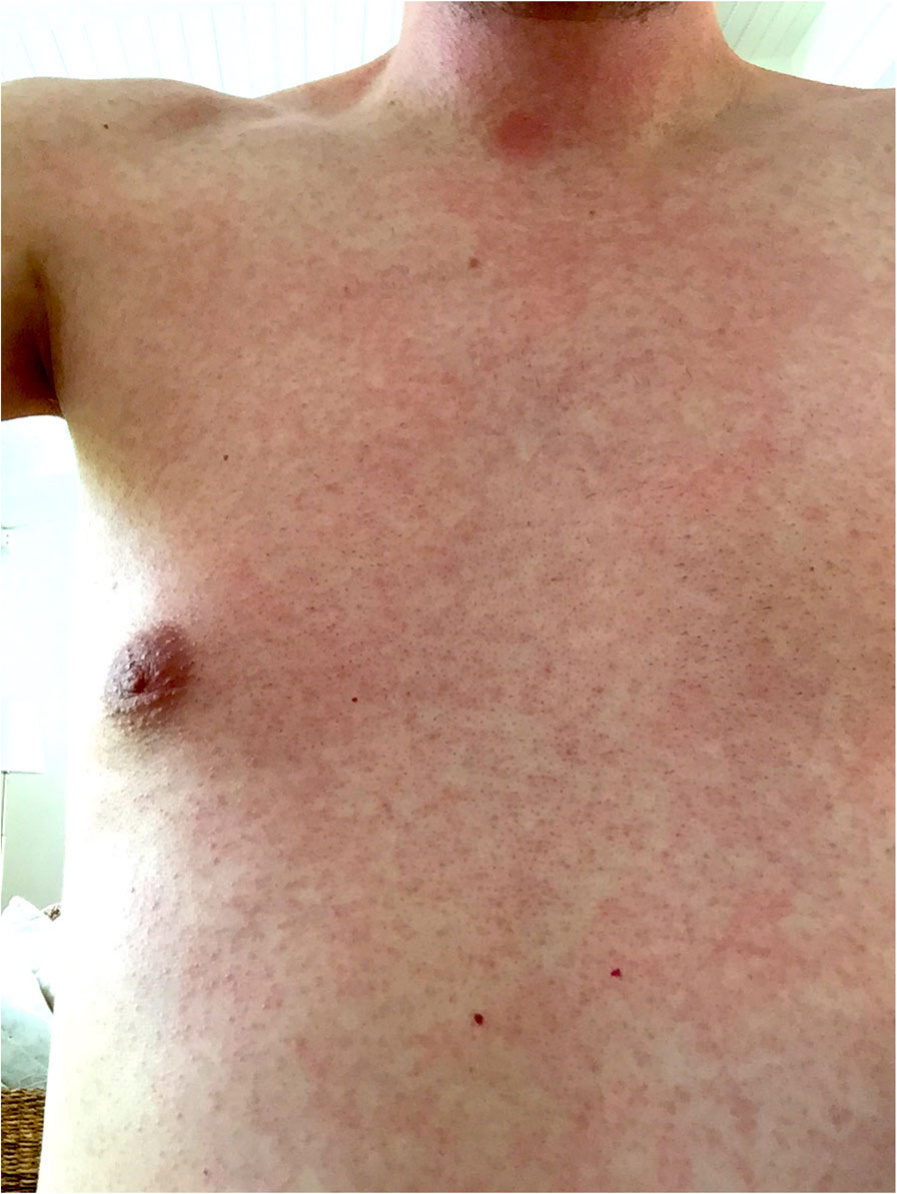
Macular rash on day three after symptom onset. Source: the photograph has been kindly provided by the patient

A 41-year-old male patient presented at the outpatient clinic of the Department for Infectious Diseases and Tropical Medicine on 30 May 2016, one day after returning from Martinique where he had spent a two weeks holiday with his wife and his three children. The symptom onset was 6 days prior to presentation, still in Martinique and started with malaise and fever. A diffuse macular rash of the entire body (Fig. 1) developed on the following day, accompanied by fatigue, subjective hyperthermia, sweating, severe retro-orbital pain without signs of conjunctivitis, general myalgia, painfully enlarged occipital lymph nodes and pain in the elbow joints.

The travel history revealed previous trips to Vietnam in 2006, India in 2012, Turkey in 2014, and Guadeloupe in 2015, without any medical history suggestive of a previous flavivirus infection. The further previous history revealed in addition to routine immunizations vaccinations against tick borne encephalitis virus and yellow fever virus. Notably, the patient had been vasectomized for family planning purposes in 2015. Physical examination revealed a patient in only slightly reduced general condition and good nutritional status with an already pale but still clearly visible macular rash. An enlarged lymph node was palpable and tender in the right occipital area. The auricular temperature was 37.4 °C. The remaining findings of the physical examination were not remarkable. On digital palpation of the prostate no abnormal finding could be noted. Ultrasound investigation of the abdomen revealed no pathological findings. The neurological examination yielded no remarkable results.

As a suspect case of ZIKV infection according to the case definition of WHO [12] the patient was included, after signing an informed consent form, in an ongoing study of ZIKV infections (“Diagnostics of Zika virus Infection in Different Body Fluids”) conducted jointly between the Department for Infectious Diseases and Tropical Medicine and the Department of Virology and Rickettsiology of the Bundeswehr Institute of Microbiology (BwIM). The study has been granted ethical clearance by the Ethics Committee of the Ludwig-Maximilians-Universität, Munich (opinion number 223–16).

### Clinical course

The patient recovered without receiving any specific treatment. All symptoms resolved completely by 10 June, besides a markedly depressed mood that lasted until the end of June.

### Laboratory investigations

Clinical samples were collected 6, 9 and 14 days after disease onset and from then on a weekly basis until 91 days after symptom onset. After day 91 the visit intervals were prolonged due to periods of absence of the patient. He was seen again on day 101, 112 and 119. Specimens were transported to BwIM, and stored at −80 °C prior to laboratory investigation.

In order to verify azoospermia in the vasectomized patient, examination of ejaculate by microscopy was conducted.

Nucleic acid preparation: Viral RNA was extracted using the QIAamp viral RNA Mini Kit (Qiagen, Hilden, Germany) according to the manufacturer’s instructions. Prior to extraction, ejaculate, EDTA whole blood and saliva specimens were diluted 1:2 in nuclease free water to avoid PCR inhibition. PCR: All specimens were investigated for the direct detection of ZIKV using the commercially available real-time reverse transcriptase assay RealStar Zika RT PCR Kit (Altona, Hamburg, Germany). Coinfection with chikungunya virus (CHIKV) or dengue virus in the patient was excluded by testing the serum sample six days after symptom onset by means of the commercially available real-time reverse transcriptase assays RealStar Dengue RT PCR Kit and RealStar Chikungunya RT PCR Kit (both purchased from Altona, Hamburg, Germany), respectively, according to the manufacturer’s instructions.

### Serology

Serological testing was conducted in all serum samples using commercially available immunofluorescence tests for the detection of IgG and IgM antibodies against ZIKV, DENV, CHIKV, Japanese encephalitis virus (JEV), tick-borne encephalitis virus (TBEV), West Nile virus (WNV) and yellow fever virus (YFV) (Euroimmun, Lübeck, Germany). Twofold dilutions of sera were subjected to endpoint titration with starting dilutions of 1:10, in order to determine IgM and IgG titers.

### Cell culture

Cultural isolation in 80% confluent monolayers of African Green Monkey kidney fibroblasts (Vero B4) and *Aedes albopictus* cells (C6/36) was attempted for urine, ejaculate, EDTA whole blood and saliva specimens. Samples were diluted 1:10 in minimal essential medium (MEM) (Life Technologies, Carlsbad, California, USA). Inocula of 500 μl each were added to 25 cm^2^ tissue culture flasks, incubated at 37 °C (Vero) or 28 °C (C6/36) for 1 h, then removed and replaced by 5 ml MEM containing 3% fetal bovine serum, 1% non-essential amino acids (Life Technologies, Carlsbad, California, USA) and 3% Antibiotics/Antimycotics Solution (Life Technologies, Carlsbad, California, USA). Cultures were incubated at 37 °C or 28 °C for 7 days and then passaged using the procedure described above.

All cultures were observed daily for cytopathic effects. Also, viral growth was confirmed by Cycle Threshold (CT) value decrease in real-time reverse transcriptase-PCR assay between day 0 and day 7 of infection.

### Sequence analysis

In order to subtype the strain of ZIKV in this patient sequences of two genomic regions (non-structural proteins 2b and 3 (NS2b/NS3) and 5′ untranslated region (5UTR)) were investigated.

PCR products were obtained by conventional reverse transcriptase-PCR. Briefly, fragments of 772 base pairs and 345 nucleotides size were amplified from the extracted nucleic acid using the primers Z4360 F 5′- CTCTgTACACTCCATCTgTggTC-3′ and Z5108 R 5′- gACAgTTAgCTgCTTCTTCTTCAg −3′(NS2b/NS3) and the primers 5UTR F 5′- TgTTgATCTgTgTgAATCAgACTg −3′and 5UTR R1c 5′-CTCTTCTAgATCTCCgTgCTTCAC −3′ (5UTR), respectively. All protocols were carried out using a superscript III high fidelity kit (Life Technologies) and an identical thermoprofile. Briefly, 5 μl RNA, 0.4 μM forward and reverse primers, 1 μl enzyme mix, 1 x buffer and 2.4 mM MgSO4 were mixed and PCR-grade water was added to a final volume of 50 μl/reaction. The thermoprofile consisted of 20 min reverse transcription at 50 °C and an initial denaturation for 2 min at 95 °C, followed by 40 cycles each featuring 15 s at 95 °C, 30 s at 58 °C and 30 s at 68 °C and a final extension step for 7 min at 68 °C. The obtained PCR fragments were analysed by Sanger sequencing conducted by an external contractor (GATC, Konstanz, Germany). Phylogenetic tree analysis was performed using the Maximum Likelihood Method based on the Tamura-Nei model and 1000 bootstrap replicates with Mega 5.0 [13]. Identical clusters and highly similar confidence values were obtained by Maximum Parsimony method and Neighbour-Joining method.

Routine laboratory tests including blood count and clinical chemistry parameters as well as serological differential diagnostics for common infectious diseases were performed at the laboratory of the Department for Infectious Diseases and Tropical Medicine and an external sub-contractor.

## Results

Besides slightly elevated transaminases and C-reactive protein as well as mild micro-haematuria routine laboratory investigations revealed no pathological findings. The blood group was determined as group A, Rhesus factor positive. The Prostate Specific Antigen level in serum was within the limits of normal.

In a native ejaculate specimen and after centrifugation no spermatocytes could be identified by sampling 2x200 visual fields, indicating a successful vasectomy. There were 0.7 × 10^6^ round cells per ml ejaculate (these consist usually of immature spermatocytes and leucocytes in a non-vasectomized male), and among these 0.05 × 10^6^ peroxidase positive cells per ml (indicating neutrophil leukocytes). By microscopy neither immature spermatocytes nor epithelial cells were found by an experienced examiner.

### Molecular diagnostics

Initially, only a urine and a serum sample were tested for ZIKV, DENV and CHIKV. The urine tested PCR-positive for ZIKV on day 6 after symptom onset, while the serum sample was found to be PCR negative for ZIKV, DENV and CHIKV. A concurrent DENV and CHIKV infection was thus excluded.

**Fig. 2:**
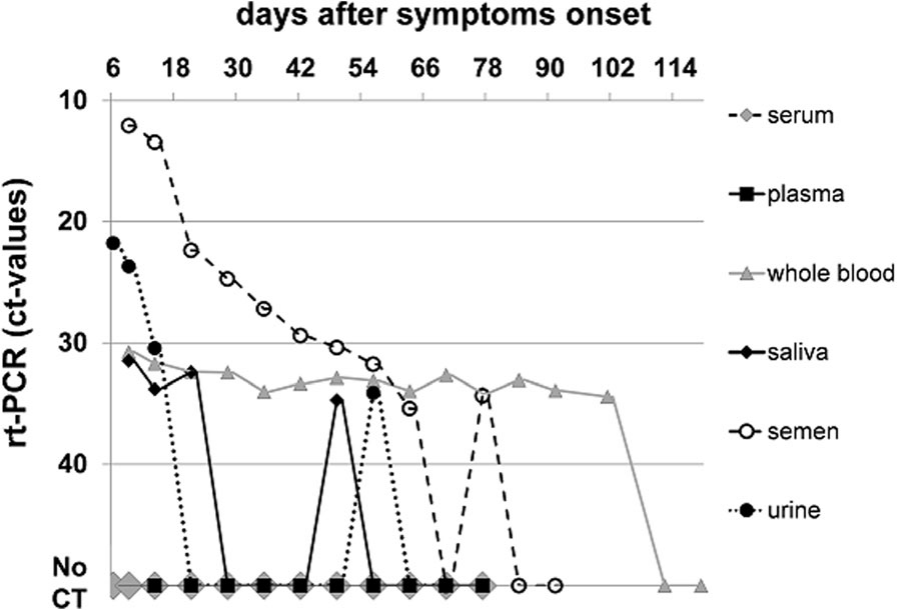
Graph of PCR CT-values in different sample types

Weekly follow up specimens from day 9 to day 77 included ejaculate, saliva and EDTA whole blood, in addition to serum and urine. Urine and saliva were found to be positive until day 14 and 21, respectively; both sample types were again tested positive on a single occasion on day 49 (saliva) and day 56 (urine). These resurgences were not accompanied by clinical symptoms in the patient. After day 77 only collection of ejaculate and whole blood samples was continued. Ejaculate and whole blood were tested positive for ZIKV by PCR until day 77 and 101 respectively. Collection of ejaculate samples was discontinued after day 91 when the second consecutive negative result was obtained. The follow-up period was closed on day 119 when for the whole blood samples the second consecutive negative result was obtained (Fig. 2).

Serum and plasma samples did not yield any positive PCR-results throughout the investigation period. Detailed results are displayed in Table 1.

**Table 1:** Sample types and test results of laboratory examinations for ZIKV up to day 119 post symptom onset

	PCR Results (CT-values)				Cell Culture	Serology Titers	
Days after symptom onset	Urine	Saliva	Whole Blood	Ejaculate	Ejaculate	IgM	IgG
day 6	**pos (21.75)**	nd	nd	nd	nd	**160**	**640**
day 9	**pos (23.67)**	**pos (31.47)**	**pos (30.75)**	**pos (12.07)**	**pos**	**640**	**5120**
day 14	**pos (30.44)**	**pos (33.79)**	**pos (31.71)**	**pos (13.45)**	neg	**320**	**10240**
day 21	neg	**pos (32.41)**	**pos (32.39)**	**pos (22.36)**	**pos**	**160**	**5120**
day 28	neg	neg	**pos (32.45)**	**pos (24.72)**	neg	**40**	**2560**
day 35	neg	neg	**pos (34.01)**	**pos (27.19)**	neg	**10**	**2560**
day 42	neg	neg	**pos (33.35)**	**pos (29.40)**	neg	**10**	**2560**
day 49	neg	**pos (32.87)**	**pos (34.70)**	**pos (30.39)**	nd	neg	**2560**
day 56	**pos (34.1)**	neg	**pos (33.1)**	**pos (31.77)**	nd	neg	**2560**
day 63	neg	neg	**pos (33.95)**	**pos (35.40)**	nd	neg	**2560**
day 70	neg	neg	**pos (32.66)**	neg	nd	neg	**2560**
day 77	nd	neg	**pos (34.14)**	**pos (34.34)**	nd	neg	**2560**
day 84	nd	nd	**pos (33.05)**	neg	nd	neg	**1280**
day 91	nd	nd	**pos (33.92)**	neg	nd	nd	nd
day 101	nd	nd	**pos (34.42)**	nd	nd	nd	nd
day 112	nd	nd	neg	nd	nd	neg	**1280**
day 119	nd	nd	neg	nd	nd	nd	nd

ZIKV positive test results are typed in bold face. Only test results for sample types that tested positive at least once during the sampling period are shown. *pos* positive, *neg* negative, *nd* not determined

### Serology

**Fig. 3:**
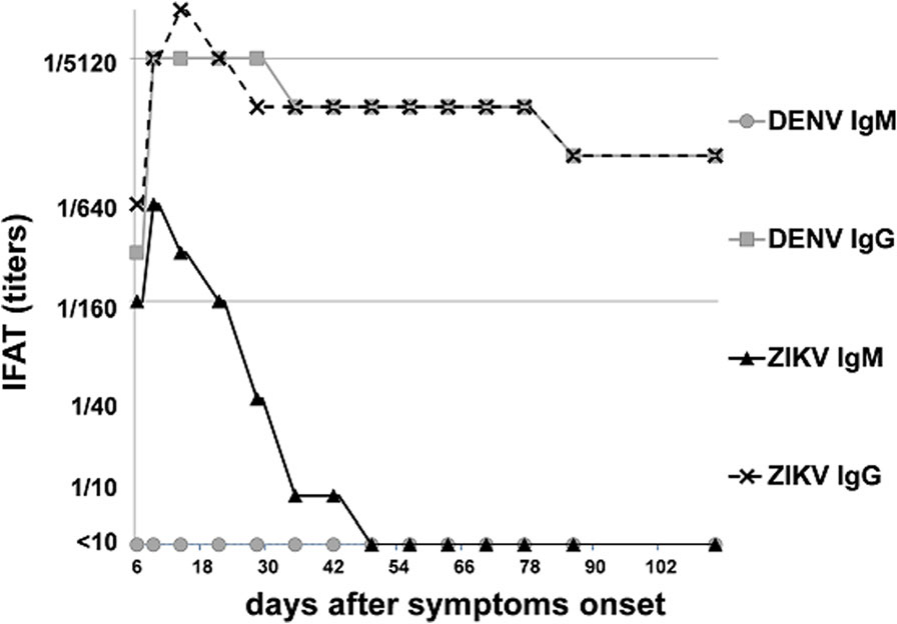
Course of IgM and IgG response against ZIKV and DENV. IgM antibodies were exclusively reactive against ZIKV antigen while for IgG a broad reactivity against other flaviviruses was observed (data only shown for DENV)

IgM antibodies were exclusively reactive against ZIKV antigen while for IgG a broad reactivity with other flaviviruses (DENV, JEV, TBEV, WNV and YFV) was observed. No IgM or IgG antibodies against CHIKV were observed throughout the whole investigation period. Anti-ZIKV IgM antibodies reached their peak on day 9 and turned undetectable on day 49, while anti-ZIKV IgG antibodies reached their peak on day 14 and remained detectable at a stable titer level until the end of the observed period. The serological course of ZIKV and DENV IgM and IgG is depicted in Fig. 3.

### Virus isolation

A ZIKV strain was isolated in both cell lines from an ejaculate sample collected on day 9, but only in *Aedes albopictus* cells in an ejaculate sample from day 21 post symptom onset. The strain was designated TM 100.16 ZIKV Martinique. Attempts for virus isolation from other sample types remained unsuccessful.

### Sequence analysis of the isolated ZIKV strain

**Fig. 4:**
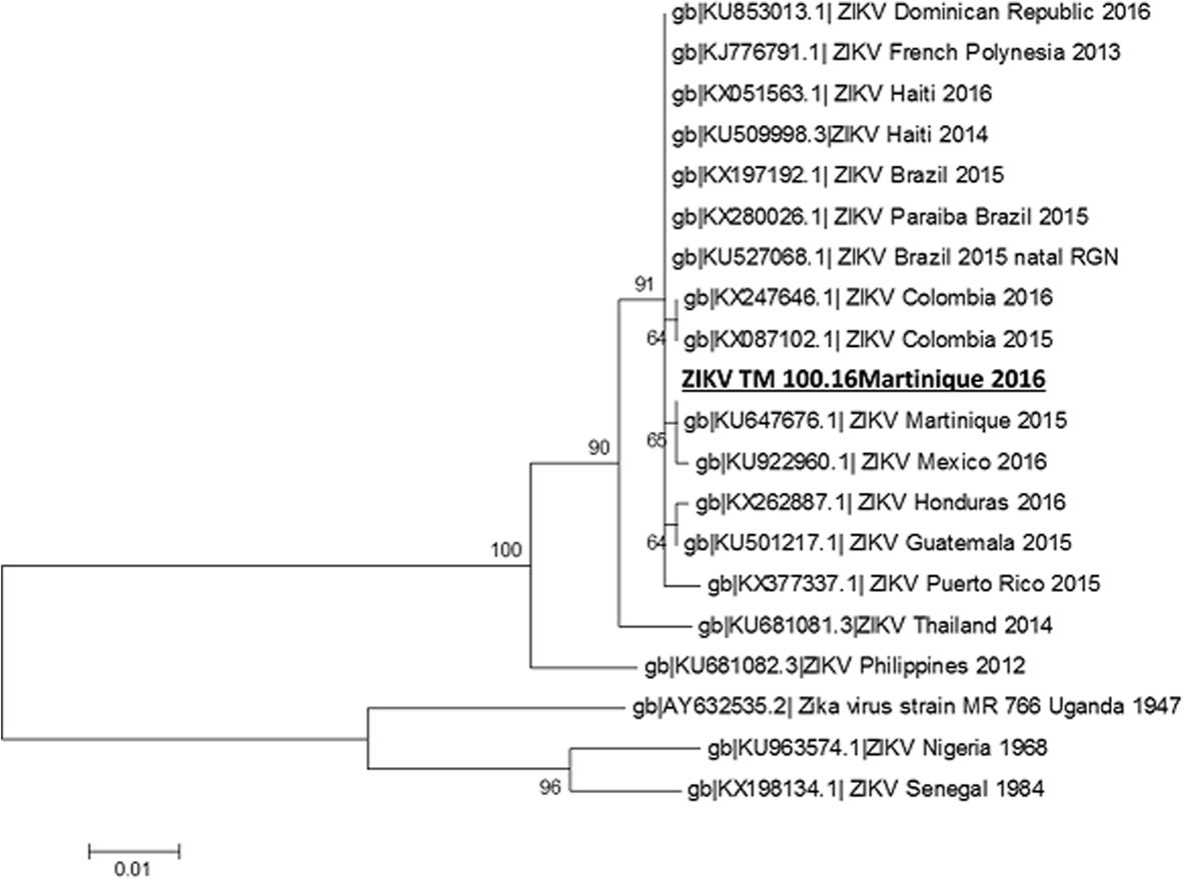
Phylogenetic tree analysis for NS2b and NS3 of ZIKV strain TM 100.16. Phylogenetic tree analysis of a 772 nucleotide partial segment of NS2b and NS3 by Maximum Likelihood method based on the Tamura-Nei model and 1000 bootstrap replicates with Mega 5.0; the patient isolate TM 100.16 ZIKV Martinique is highlighted in bold (scale bar indicates number of nucleotide substitutions per site)

**Fig. 5:**
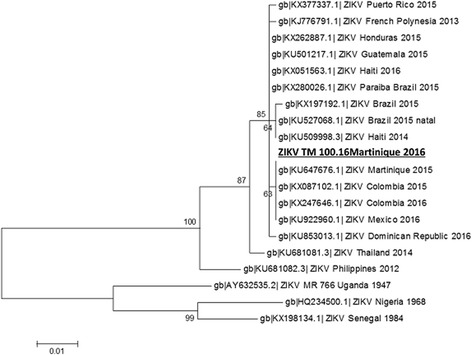
Phylogenetic tree analysis for the 5′untranslated region of ZIKV strain TM 100.16. Phylogenetic tree analysis of a 608 nucleotide partial segment of the 5′untranslated region by Maximum Likelihood method based on the Tamura-Nei model and 1000 bootstrap replicates with Mega 5.0; the isolate TM 100.16 ZIKV Martinique is highlighted in bold (scale bar indicates number of nucleotide substitutions per site)

Sequencing of PCR amplicons of the ZIKV strain isolated from the patient revealed an Asian strain of ZIKV. The phylogenetic tree that was constructed for non-structural proteins 2b and 3 (Fig. 4) and for the 5′ untranslated region (Fig. 5) by sequence alignment with reference data located the identified strain in close proximity to strains already identified in 2015 in Martinique, and in 2016 in Colombia and Mexico.

## Conclusions

The ZIKV epidemic in Latin America is also affecting German travellers returning from regions with circulating ZIKV infections. We are reporting a profound follow-up investigation in a vasectomized patient with ZIKV infection providing a detailed dataset of serological as well as molecular parameters elucidating the kinetics of ZIKV RNA presence in various sample types. We also contribute the results of our isolation attempts and the sequence data of a new ZIKV strain. ZIKV RNA was detected over a long period of time up to 77 days post symptom onset in ejaculate and up to 101 days in whole blood samples of the patient. The prolonged detectability of viral RNA has recently been reported for some sample types such as ejaculate [7] saliva [14] or whole blood [11] by other authors, as well as the possibility of a resurgence of positive PCR results in saliva after prior conversion to negative [14]. The intermittent detection of ZIKV RNA in urine as demonstrated in this study might be interpreted as contamination due to the very close temporal relation of the collection of urine and ejaculate samples.

Notably, the patient has been successfully vasectomized in 2015. Thus, the persistence of ZIKV in the urogenital tract in males, or at least its location of shedding, which is still poorly understood in humans, can be narrowed down in the patient described in this study to the compartments of Cowper’s glands, the prostate gland and seminal vesicles; the latter two contributing the largest fractions to the ejaculate volume. The low CT-values found in ejaculate specimens and the prolonged detectability of viable ZIKV in this body fluid indicate that ZIKV might not only persist, but also replicate in a yet not clearly defined compartment of the male urogenital tract. ZIKV persistence in patients with clinical signs of prostatitis has been reported elsewhere [15]. However, our patient did not show any clinical signs of inflammation in the urogenital tract, indicating that this is not a prerequisite for replication of ZIKV in this compartment. A recent report on another vasectomized patient returning from the Maldives also found ZIKV in ejaculate samples. There, a potentially sexual transmission to a partner has been discussed. However, the possibility of a mosquito-borne transmission in the wife cannot be entirely ruled out as she had accompanied the index patient on the visit to the Maldives, and her symptoms started only 18 days after their return [16]. Mansuy et al. demonstrated the presence of ZIKV particles in the heads of spermatocytes [17]. In an attempt to identify the compartment in ejaculate harbouring ZIKV particles, Barzon et al. investigated both seminal plasma and the cellular compartment of ejaculate samples positive for ZIKV. They found ZIKV nucleic acids exclusively in the cellular fraction [18]. However, in this present study on a successfully vasectomized patient spermatocytes could be excluded as carriers of viral particles, it therefore may be speculated that other cellular elements may be involved, namely the round and peroxidase positive cells that were found in the ejaculate of our patient. These cells are either immature sperm forms (e.g. spermatocytes) or inflammatory cells (like neutrophils, lymphocytes, macrophages and epithelial cells) [19]. In this patient presence of both immature spermatocytes and epithelial cells could be ruled out, therefore at large the cellular compartment of the ejaculate consisted of leukocytes. Also, the location of ZIKV particles in the male genitourinary tract might be variable and even the possibility that ZIKV might be capable to attach to a diverse number of cell types and thus be distributed in the organism has to be considered. In the future, investigations are needed to shed light on the anatomical location of viral persistence and the exact compartment in ejaculate that is responsible for the shedding and carriage of viral particles.

The successful cell-cultural isolation of ZIKV from the ejaculate in this patient demonstrated viability of the virus until at least 21 days post symptom onset. The failure of cell cultural isolation beyond this point of time might have been caused by deterioration of the viability of the virus due to low pH or other specimen-dependent influencing factors, as recently proposed by other authors [20, 21]. Thus, the potential of sexual transmission for a much longer period in this patient cannot be ruled out.

A prolonged isolation of viral RNA in whole blood samples has previously been proven for other flaviviruses, e.g. by Klungthong et al. for dengue virus [9] or by Lanteri et al. for West Nile virus [10]. In the latter publication, a correlation between blood group A and higher viral loads has been claimed, possibly indicating an attachment of viral particles to erythrocyte membranes. Our patient has also blood group A.

Our results also indicate that ZIKV particles might be bound to cellular structures, because serum samples recovered from the same venepuncture as well as plasma samples obtained after centrifugation of a ZIKV positive whole blood sample were tested negative for ZIKV RNA. To clarify the exact localization of ZIKV particles in the cell compartment in blood, further analyses of distinct fractions of whole blood including the buffy coat, as well as imaging techniques such as electron microscopy and fluorescence in situ hybridization techniques in additional patients are planned.

Our data provide some evidence that during the late course of the disease, whole blood may be the type of sample giving the best diagnostic sensitivity. Current guidelines in vigour are suggesting collection of serum samples until day 7 after symptom onset, and of urine until day 21 for direct detection of viral RNA. Investigations at later time points are based on serological, thus indirect detection methods. Based on findings of different groups that parallel our results, sample types have been suggested for later direct detection of ZIKV RNA, such as saliva or whole blood [11, 14]. Based on the results of this study, whole blood seems to be the most promising candidate for late detection of viral particles in patients, especially, when IgM antibodies are no longer detectable. Ejaculate is another option in male patients and also allows late direct diagnosis, but is less suitable in routine diagnostics due to the restriction to male patients and the effort of specimen collection Therefore, it should be mainly considered as diagnostic sample for certain clinical settings, such as fertility clinics or couples wishing to become pregnant that travel frequently to or are residing in endemic regions. In addition, examination of ejaculate samples may be a means to rule out further infectiousness in male patients originating from non-endemic regions after having completed the recommended six months of sexual barrier protection, especially in couples that wish to conceive.

In terms of infection prevention, different current national and international guidelines have adapted their recommendations concerning sexual barrier protection for persons returning from ZIKV endemic countries. The periods of recommended use of barrier protection, such as condoms, is currently set to six months after return from an endemic region. These time frames are based on reports of observations of direct ZIKV detection in ejaculate, such as in this patient, rounded up by a safety time buffer [22, 23].

Saliva samples seem to be another suitable routine specimen type in the acute phase of the infection, with a slightly longer persistence time than in urine, but seem to remain positive for shorter periods than whole blood specimens. Saliva specimens may particularly be considered in situations where processing of blood samples may be difficult, such as in paediatric populations or in screening activities either of large populations or in low resource settings.

The long duration of detectability of viral RNA, and possibly also of viable and therefore infectious virions in whole blood presents a challenge to medical uses of human blood products. A risk of transmission of ZIKV by blood-donors as well as platelet donors has been reported recently [24]. It appears reasonable to suggest that blood donors need to be screened for potential exposure to ZIKV, and individuals fulfilling criteria of potential exposure (such as travelling in ZIKV endemic countries or having had sexual contact to a person that has travelled to ZIKV endemic countries within the past six months) should have a whole blood sample tested directly by PCR as well as indirectly by serological testing prior to donation.

The serological profile in our patient revealed an expectable dynamic with an earlier peak in anti-ZIKV IgM antibodies as compared to anti-ZIKV IgG antibodies. On day 49, anti-ZIKV IgM antibodies were not detectable any more. As shown in Fig. 3, anti-DENV IgG antibodies also showed an early increase, while at no time point anti-DENV IgM antibodies were detectable. The IgG and IgM antibodies of the other investigated flaviviruses (JEV, TBEV, WNV and YFV) showed profiles similar to DENV. Since the patient had no medical history suggestive of a previous flavivirus infection but has been vaccinated against TBEV and YFV several years ago, it may be argued whether the IgG antibody profiles in assays directed at flaviviruses other than ZIKV developed due to cross-reactions between anti-ZIKV antibodies and the used immunofluorescence based serological assays, or whether an immunological memory that had been primed by previous vaccinations or unrecognized flavivirus infections underwent a bystander activation in the current course of an acute ZIKV infection.

In summary, the results of this case enhance the knowledge about long-term kinetics of direct and indirect ZIKV detection methods and moreover about possible locations for persistence of ZIKV in whole blood and the male reproductive tract.

## Abbreviations

CHIKV: Chikungunya virus
CT: Cycle threshold
DENV: Dengue virus
JEV: Japanese encephalitis virus
PCR: Polymerase chain reaction
TBEV: Tick-borne encephalitis virus
WNV: West Nile virus
YFV: Yellow fever virus
ZIKV: Zika virus
